# Effect of pressure and padding on motion artifact of textile electrodes

**DOI:** 10.1186/1475-925X-12-26

**Published:** 2013-04-08

**Authors:** Alper Cömert, Markku Honkala, Jari Hyttinen

**Affiliations:** 1 Department of Biomedical Engineering, Tampere University of Technology, Tampere, Finland; 2 Institute of Biosciences and Medical Technology, Tampere, Finland; 3 Department of Material Science, Tampere University of Technology, Tampere, Finland

**Keywords:** Textile electrodes, ECG, Motion artifact, Electrode-skin interface impedance

## Abstract

**Background:**

With the aging population and rising healthcare costs, wearable monitoring is gaining importance. The motion artifact affecting dry electrodes is one of the main challenges preventing the widespread use of wearable monitoring systems. In this paper we investigate the motion artifact and ways of making a textile electrode more resilient against motion artifact. Our aim is to study the effects of the pressure exerted onto the electrode, and the effects of inserting padding between the applied pressure and the electrode.

**Method:**

We measure real time electrode-skin interface impedance, ECG from two channels, the motion artifact related surface potential, and exerted pressure during controlled motion by a measurement setup designed to estimate the relation of motion artifact to the signals. We use different foam padding materials with various mechanical properties and apply electrode pressures between 5 and 25 mmHg to understand their effect. A QRS and noise detection algorithm based on a modified Pan-Tompkins QRS detection algorithm estimates the electrode behaviour in respect to the motion artifact from two channels; one dominated by the motion artifact and one containing both the motion artifact and the ECG. This procedure enables us to quantify a given setup’s susceptibility to the motion artifact.

**Results:**

Pressure is found to strongly affect signal quality as is the use of padding. In general, the paddings reduce the motion artifact. However the shape and frequency components of the motion artifact vary for different paddings, and their material and physical properties. Electrode impedance at 100 kHz correlates in some cases with the motion artifact but it is not a good predictor of the motion artifact.

**Conclusion:**

From the results of this study, guidelines for improving electrode design regarding padding and pressure can be formulated as paddings are a necessary part of the system for reducing the motion artifact, and further, their effect maximises between 15 mmHg and 20 mmHg of exerted pressure. In addition, we present new methods for evaluating electrode sensitivity to motion, utilizing the detection of noise peaks that fall into the same frequency band as R-peaks.

## Background

In this work we aim to shed light onto possibilities to reduce the textile electrode’s motion artifact by the use of applied pressure, and how this pressure, together with possible paddings placed on the electrode, affects the stability of the electrode-skin interface. In the diagnosis and treatment of cardiovascular disease, the main tool is the ECG (electrocardiogram), and is mostly done by medical personnel. This specific need for medical personnel to be present almost at each instance of an ECG increases the cost per assessment. Considering that cardiovascular disease is the main cause of death in Western societies [1] and the prevalence of the ECG, it is clear that it imposes a heavy cost and personnel load on the healthcare systems.

Interest in the mobile monitoring of the ECG has been rising in the past decades, for diagnosis, as well as screening, risk assessment, prevention and rehabilitation. Mobile monitoring provides the patient the freedom to move and reduces the need of near constant supervision after an initial set up by medical personnel. Holter Monitors have existed for quite some time, monitoring ECG away from the hospital using commercial ECG electrodes.

One issue with current mobile systems using commercial gelled electrodes is the electrode-skin interface over a long time period. The electrode gel used for improving the contact to skin may dry over time, causing loss of signal quality; the glue holding the electrode in place may come off, or may cause skin irritation [2]. The skin abrasion effect wanes after approximately 24 hours [3]. The cabling may cause discomfort, and may affect signal quality under certain conditions. Wearable heart rate monitors that solve these problems and enable long term mobile monitoring are on the market. Few examples would be Polar heart rate monitors (Polar Electro, Kempele, Finland), Suunto sports watches (Suunto, Vantaa, Finland), and Adidas miCoach heart rate monitor module (Adidas, Herzogenaurach, Germany) whose chest belts are supplied by Clothing + (Kempele Finland). However, using electrodes attached to a chest belt, their scope does not cover standard ECG leads.

The challenge in the case of wearable medical ECG is to design electrodes that are part of a garment that provides reliable ECG monitoring over time. These electrodes need to have reliable signal quality for long term monitoring including various conditions of daily and sports activity, cause minimal discomfort to the wearer, and be reused by the wearer without the presence of medical personnel, except maybe for an initial setup. Textile electrodes made using silver yarn, having acceptable electrical properties, being antibacterial, unobtrusive and washable could be integrated into clothing. If textile cables are used for electrical connections, a truly wearable system could be achieved.

The main challenge with implementing textile electrodes is that they basically act as dry electrodes. No electrode gel is used between the electrode and skin. Thus, in the absence of sweat, the contact impedance between electrode and skin for dry electrodes is higher than traditional commercial electrodes. Also, they are not fixed on to the skin via a sticky membrane, necessitating the use of other attachment methods. Due to these two factors, dry textile electrodes are especially motion artifact prone, and this susceptibility to the motion artifact is the main reason they have not yet been widely implemented in available systems.

Various projects that aim to integrate the electrodes into clothing for daily use, such as the WEALTHY project [4], the MyHeart project [5], the VTAMN project [6], the LifeShirt (Vivometrics Inc., Ventura, USA), the CorusFit vest (CorusFit OY, Jyväskylä, Finland) [7], and others [8-11] have produced promising results. In these and other projects, the implementation of textile electrodes as dry electrodes [6,8,9,12], textile electrodes together with various gels to improve the electrode-skin contact [4,13], as well as dry contact electrodes not made of textile [14,15] have been investigated with varying but mostly positive results.

Adaptive filtering guided by the electrode-skin interface impedance and acceleration measurements are successfully implemented in reducing the susceptibility of the dry electrodes to the motion artifact [16-20], resulting in reducing the motion artifact, increasing the signal quality and improving the QRS detection. For example Kirst reports a 35% decrease in false QRS detection, using discrete wavelet transforms together with electrode-skin interface impedance [21]. Comparable results are obtained by the implementation of active electrodes [22] and using independent component analysis for artifact removal [23,24]. Using optical devices to gauge skin stretch and using stretch sensors and bend sensors to asses electrode motion decrease the motion artifact [25,26], albeit to a lesser extent than the previous methods.

In this study we aim to gain understanding on the relation between pressures exerted on the electrode and the observed motion artifact, and on the possible effect of the use of padding between the electrode and the clothing fabric for various pressures. Related to this we investigate if different padding materials have effect on various measured parameters related to the ECG, motion artifact and electrode-skin interface impedance. To obtain these parameters we measure the ECG, motion artifact related surface potential, real time electrode-skin interface impedance and exerted pressure, during controlled motion and calculate various parameters related to these signals. The overall study is done by using a novel method using two measurement channels designed so that both channels are exposed to the motion artifact, but one channel has a strong ECG component while the other channel has a negligible ECG component. A third channel, derived from the former two, provides an ECG signal that is not susceptible to the specific motion artifact. Results of this study can then be used in system that could monitor ECG in sports, daily life, and in low risk hospital stays, providing a stable ECG even during activity.

### Origins of the motion artifact and the electrode-skin interface model

**Figure 1:**
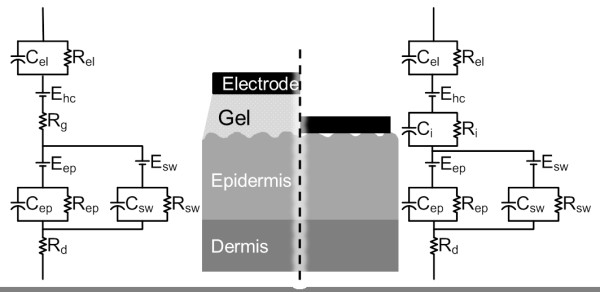
**The electrode-skin interface model for gelled electrodes and dry electrodes.** The diagram is modified from [29] and [30]. The electrical model for gelled electrodes is on the left, the electrical model for the dry electrodes is on the right.

The most widely accepted explanation of the origins of the motion artifact comes from Webster [3,27] describing that the main component of the motion artifact is the change in the potential across the epidermis. This potential is shown as E_ep_ on the skin model in Figure 1 and its amplitude changes upon skin deformation by lateral stretching or applying vertical pressure. The changing impedance of the epidermis, due to changing current pathways, is mentioned as a minor effect in the motion artifact.

In the case of dry electrodes, this explanation for the cause of the motion artifact, given for gelled electrodes, may be insufficient. Electrode gel is very stable against lateral and vertical changes in its geometry and thus the electrode-skin interface is not affected much by motion. For dry electrodes, the conductive gel layer does not exist and the contact area may change considerably with applied pressure and motion, affecting the electrical properties of the electrode-skin interface [28]. Since the interface layer is a thin layer of skin humidity, small changes in the electrode location will cause large disturbances in the ionic concentration close to the electrode, in turn changing the half-cell potential. All these changes are observed as motion artifact on the measured biopotential. Thus, these factors have to be included in the list of possible causes for the motion artifact.

When the electrode is in contact with an electrolyte, depending on the electrode material and the electrolyte composition, the ion concentration forms a gradient. This difference in the ion concentration is seen as a voltage across the electrode-electrolyte interface and is called the half-cell potential. The electrolyte, having high conductivity, acts as a resistor. To complete the model for the electrode-skin interface, the layers of skin have to be taken into account. The outmost layer of the skin, called the epidermis, consists of a layer of dead cells and two layers that take part in the production of these cells that form the surface of the skin. An ion gradient exists across this skin layer, creating a potential difference. This level can be modeled as a voltage source in series with a parallel circuit consisting of a resistor and capacitor. Additionally, in the presence of sweating, the sweat ducts and the included sweat are an effective current pathway, and dominate the current pathway as sweat is a better conductor than the epidermis layer. Under the epidermis lies the dermis and subcutaneous tissue, which can be modeled as a simple resistor [29].

The electrode-skin interface model is depicted in Figure 1, E_hc_ is the half-cell potential, C_el_ and R_el_ are the capacitance and resistance of the electrode material and electrode electrolyte interface, R_g_ is the resistance of the electrolyte. The potential across the epidermis due to the ionic difference between the dead cell layer and the underlying layers is named as E_ep_, and the epidermis’ capacitance and resistance as C_ep_ and R_ep_. The dermis is noted as R_d_. In the presence of sweat, the sweat ducts start acting as a current pathway, and their contribution is modeled as E_sw_ in series with the parallel R_sw_ and C_sw_.

For dry electrodes, as depicted in the model on the right in Figure 1, when no electrolyte is present, the electrode-skin contact is not uniform, with occasional air bubbles between the electrode and the skin, and the galvanic connection is mainly realized by the humidity and sweat on the skin. In this case, the presence or absence of sweat makes a big difference in the electrode-skin interface, and the electrode-skin interface is modeled by a resistor in parallel with a capacitor R_i_ and C_i_. R_i_ is generally larger that R_g_, except in the where sweat acts as an electrolyte [30].

## Methods

### Electrode and motion artifact measurement

To investigate the motion artifact and its suppression when using textile electrodes, electrodes were fabricated from the commercially available Medtex P180 (Statex Productions & Vertriebs GmbH, Bremen, Germany) warp knitted textile made of silver coated yarn. This textile is antibacterial, conductive and stretchable, and usable as electrodes since the fabric surface is highly conductive. The electrode-skin interface impedance in dry conditions is suitable for biopotential measurements. The elasticity of this textile was not used in this study.

The electrodes were connected to the measurement system using unshielded textile cables (Finn-Nauha, Haapamäki, Finland) made from 234 dtex silver plated high grade multifilament 6.6 polyamide yarn which has a 10% stretch tolerance, and is covered by non-conducting polyamide yarn encasing. The cable has a resistance of 10 ohm/m and the connectors used introduced another 6 ohm/connector, at each end of the textile cable. The setup was arranged so that there was no pull on the wires and the motion of the wires was minimized.

The electrodes were fixed onto the selected locations by elastic ribbons fastened around the respective body part. A handheld PicoPress (Microlab Electronica, Ponte S. Nicolo, and Italy) sub-bandage pressure monitor was used to measure the pressures exerted by these ribbons to press the electrode to the skin.

The measurement system for ECG and biopotentials consisted of a Biopac MP35 Data Acquisition Unit (Biopac Systems, Inc., California, USA). Its add-on, a Biopac MP150 unit connected to a Biopac EBI100C Electrobioimpedance Amplifier, capable of measuring bioimpedance at four frequencies ranging from 12.5 kHz to 100 kHz, was used for bioimpedance measurements. Data analysis was done in Matlab.

Impedance measurements were done using the bipolar electrode configuration; injecting a 400 μA 100 kHz sine wave current, and measuring the resulting voltage from the same electrodes. For the purposes of this study, the bipolar configuration was used instead of the tetra polar configuration as the bipolar configuration measures mainly the electrode impedances. With the bipolar configuration, deep tissue impedance is also measured but is low compared to the skin and electrode-skin impedances, and thus was neglected in the analysis.

### Experimental procedure

A two channel ECG was recorded from three electrodes so that both channels had the same reference electrode which was located on the lateral upper right arm location. This location was where motion artifact was introduced.

**Figure 2:**
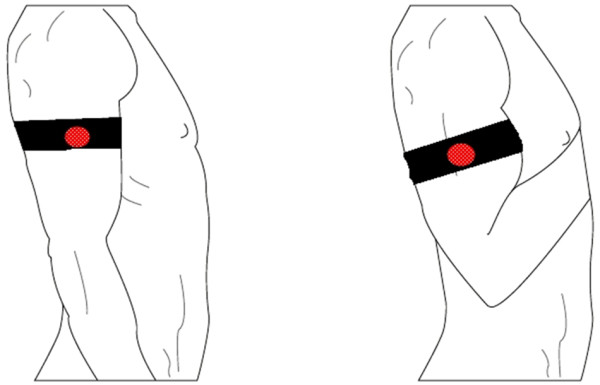
**Illustration of the motion range used in the experiments.** The figure shows the end points of motion. The forearm was moved up and down between these end points so that motion was restricted to the elbow joint. The electrode locations are shown in Figure 3.

The motion artifact measured from the upper arm was created by elbow flexion and consecutive elbow extension of an upright sitting healthy male subject. This isolated the movement only to the elbow hinge and the forearm. The motion, depicted in Figure 2, caused structural changes in the upper arm due to the biceps and triceps muscles’ deformation. A metronome set to 80 bpm was used to standardize the movement. Flexion and extension were synchronized to the metronome so that the end of flexion of the arm from the elbow was reached on one beat while the near-end of extension was reached on the next. This resulted in 40 repetitions of motion incidents per minute. The subject’s heartrate was about 65 bpm during motion. Special care was taken not to reach the end of extension as the triceps muscle’s shape change at its end of flexion was seen to be irregular and uncontrollable. The shoulder joint was kept motionless and, and the upper arm was held steady and relaxed at approximately 20 degrees laterally. This minimized the effect of shoulder motion on the motion artifact and also the deltoid muscle’s activity as EMG (electromyogram) artifact. This setup ensured a stable connection for the chest electrodes and minimized the motion artifact to negligible levels at these electrodes. Due to the extensive care taken, the motion being restricted due to the elbow joint being a hinge joint, the synchronization to the metronome, and the multiple times this motion was performed, the motion and its effect was assumed to be unvarying over the long run for the given setup.

**Figure 3:**
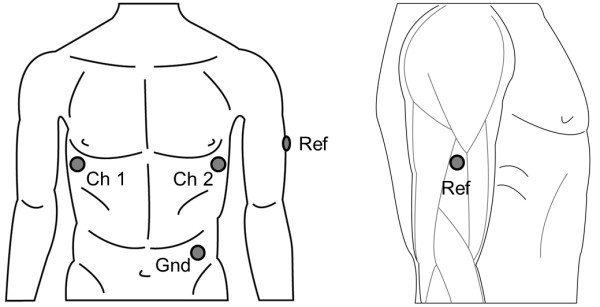
**Depiction of electrode locations.** Left hand side illustrates the locations from front; right hand side shows the detailed location for the arm electrode seen laterally. The chest electrodes are not affected by the motion described in Figure 2. The arm electrode is at a location that is minimally affected by the EMG of the arm and shoulder muscles that contract during movement.

The lateral upper right arm location to be used as reference for the two ECG channels was determined to be the best compromise between signal quality, wearing comfort and EMG interference from the distal part of the deltoid muscle, the biceps and the triceps (Figure 3). The positive electrode of the first channel was attached to the V5 location on the left chest to provide the first channel with a strong ECG component. The positive electrode of the second channel was located at the mirror location of the V5, on the right chest, in order to enable the second channel to have a very low ECG content (Figure 3). This selection of using the same electrode as a reference point for both channels, and exposing this electrode to the motion artifact, provided one signal with the motion artifact superimposed on the ECG and one signal with the motion artifact relatively unaffected by the superimposed ECG. A third signal was derived from the former two, measuring the ECG between the V5 location and its mirror location, both unaffected by the motion at the arm. This signal provided an ECG signal to be used as reference that is free of motion artifact. A fourth electrode was attached to the left hip as ground.

**Figure 4:**
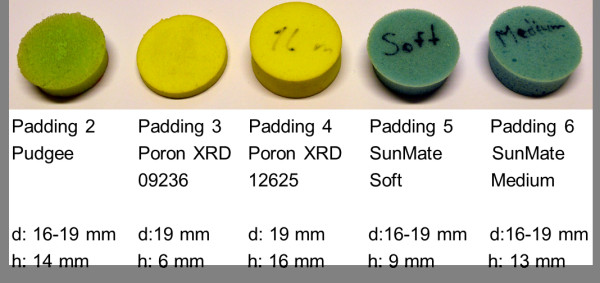
**The paddings used for the experiment.** “d” is the diameter of the padding, “h” is the height. Padding 2 is an open celled viscoelastic high density foam with gel like properties; Paddings 3 and 4 are impact protection cushions that act as memory foams in the context of this study; Paddings 5 and 6 are open celled viscoelastic polyurethane memory foams. Padding 1 is not shown because this is the name used for the case where padding was not used between the ribbon and electrode.

Out of the three textile electrodes attached to the skin by elastic ribbons, the two on the chest were held in place by an elastic ribbon around the thorax. The electrode on the upper arm was fixed by an elastic ribbon around the upper arm. In order to investigate how motion artifact and the real-time electrode-skin interface impedance is affected by the use of electrode paddings and the amount of pressure exerted on them, different types of paddings were put and tightened between the arm ribbon and the underlying electrode. The ribbon was adjusted to various pressures by changing the tightness. The pressure exerted by the ribbon was measured by the PicoPress pressure monitor on the medial part of the arm, at a mirror location to the electrode placed on the lateral side. The arm pressure under the arm ribbon was adjusted between 5 and 25 mmHg in 5 mmHg increments. The chest ribbon was held steady at 15 mmHg pressure, measured from the left lateral side of the thorax. The types of paddings tested were: two different grades of SunMate memory foam (Dynamic Systems, Inc., Leicester, USA) that is an elastomeric, open celled viscoelastic polyurethane foam; an open celled viscoelastic high density Pudgee foam (Dynamic Systems, Inc., Leicester, USA) with gel like properties; and two different grades of the Poron XRD impact protection cushion (Rogers Corporation, Rogers, USA) which act like memory foams in the context of this study. These paddings are shown in Figure 4. The textile electrodes used in the experiment were circular and had a diameter of 25 mm.

To measure the motion artifact and the ECG, the Biopac measurement system was used at the pre-set ECG setting. A pre-set impedance measurement setting was used when the Biopac was used to measure the real time electrode-skin interface from a two electrode setup. Because the intended usage for the electrodes includes the daily use by an untrained wearer, skin abrasion was omitted and cleaning done only with water and a soft towel. Due to amplifier saturation, the relatively high electrode-skin interface impedance of dry electrodes on unprepared skin prevented the simultaneous measurement of impedance and ECG from the same electrodes. A mechanical switch was designed to enable the impedance and ECG measurements to be made consecutively without tampering with the connectors, cables or electrodes or any other part of the system.

Variability between different subjects was not considered, enabling the establishment of the experimental method without the disturbance of inter subject variations. One healthy male subject was monitored in all measurements. Five different paddings and one case without the use of paddings were studied. For each of these six cases, five pressure levels were applied. This procedure was repeated six times, for a total of 180 measurements of a 30 second ECG and a consequent 30 second measurement of real-time impedance during motion. Steady state ECG and impedance were assessed for each measurement before motion was introduced for the measured signal.

The measurements were taken in a room with relatively steady temperature and humidity, due to centralized air conditioning set to circulate air at 18°C, closed windows and closed shutters.

### Data analysis

Obtained ECG was filtered using a 0.3 Hz–30 Hz band-pass filter, the impedance was filtered using a 0.3 Hz high-pass filter; and the resulting data was further analysed in Matlab. Initially, the baseline impedance, the peak-to-peak values of the impedance change and the motion artifact were analysed.

The main component in the motion artifact is the low frequency component, matching the movement frequency, which is seen like a fast baseline drift in ECG signal. This is easy to recognize and to filter out from ECG using various standard signal processing methods such as means. The bigger problem caused by the motion artifact is due the higher frequency ripples (>1 Hz) that exist within the motion artifact. To see their effect, the energies of signals containing mostly the motion artifact were calculated in various frequency bands from these signals’ power density spectrums. From these calculations, the energies of the real time impedance and the motion artifact in the frequency band between 1 Hz and 7 Hz showed the strongest relevance to the motion, and these were used in further analysis as well as one way of designating signal quality in the form of a score.

To further estimate the effect of the higher frequency components of the motion artifact on ECG signal quality, an alternate method was devised, based on the Pan-Tompkins QRS detection algorithm [31]. This devised method aimed at detecting R-peaks as well as these higher frequency noise peaks from a channel containing the motion artifact and an ECG signal. These peaks and R-peaks detected from the reference channel were used to quantify the effect the motion artifact has on the ECG signal.

Shortly in the Pan-Tompkins QRS detection algorithm, for a given channel, the derivative of a filtered ECG signal is calculated to emphasize the rising slope that leads to the R-peak, which, in the devised method also emphasizes the rising slope of high frequency (>1 Hz) noise peaks. To increase this emphasis, the derivative is squared. On the squared signal, moving window integration is done to smooth the resulting data. A threshold is selected for a moving window to detect the peaks in this window. In our method, unlike the Pan-Tompkins QRS detection algorithm, no error detection or refractory period adjustments are implemented, and noise initiated peaks are detected alongside the real R-peaks.

This peak detection method is used on two channels. The first channel is the channel across the chest, which is unaffected by the motion at the arm. Thus, the R-peaks detected from this channel, using a suitable threshold, free of noise, are used as reference. The second channel used for the peak detection is the one from the right arm to the V5 location on the left chest which includes the ECG as well as the motion artifact. For detecting the peaks of this signal, the threshold is set to a lower percentage than the threshold for the reference channel. This enables the detection of not only R-peaks and motion artifact peaks of similar amplitude, but also motion artifact peaks of lower amplitude. These lower amplitude noise peaks do not have a considerable effect on conventional R-peak detection algorithms but have a strong detrimental effect on the observed ECG characteristics like the p-wave and the t-wave. The detected peaks from this channel are matched and compared with the detected peaks from the reference channel. The peaks falling within the same 25 milliseconds window are accepted as true positives. The peaks of the reference channel not categorized as true positives are categorized as false negatives and the peaks in the noisy ECG channel that are not true positives are categorized as false positives. The noise score of padding is calculated by the formula:

$$\mathrm{Noise}\;\mathrm{Score}=\frac{\left(\mathrm{False}\;\mathrm{negatives}+\mathrm{False}\;\mathrm{positives}\right)}{\left(\mathrm{True}\;\mathrm{positives}+\mathrm{False}\;\mathrm{negatives}\right)}$$

This formula gives the number of errors per heartbeat. With this calculation, a score of 0 means no errors in R-peak detection and no detected effective motion artifact noise, while a score of 1 means that for every actual R-peak, either an R-peak is undetected, or a motion artifact peak is detected. Thus, a higher score denotes a more disruptive motion artifact.

**Figure 5:**
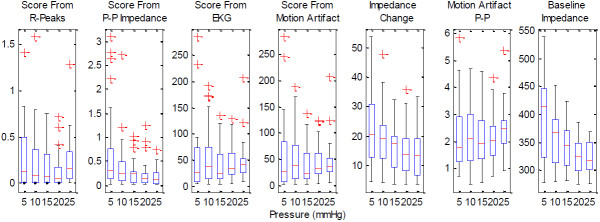
**The boxplots of various parameters obtained from the experiments utilizing the paddings.** The parameters shown are the score obtained from R-peak detection, the scores obtained from the energies of the 1 Hz–7 Hz band components of the impedance signal, ECG signal and the motion artifact, the peak-to-peak values of the motion artifact and the baseline impedance.

**Figure 6:**
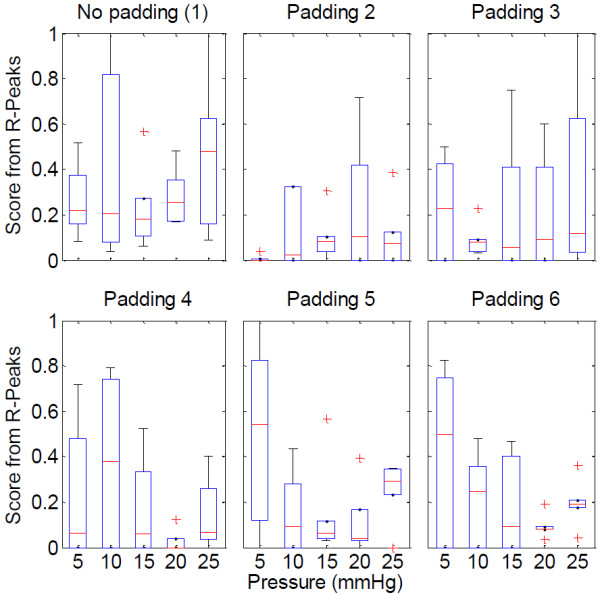
Boxplots of the scores from R-peaks for the no padding case (1) and the paddings, in relation to pressure.

The graphs presenting the results utilize the inbuilt Matlab functions. Figures 5 and 6 use the boxplot function that plots the median as the centre, the 25^th^ and 75^th^ percentiles of the data, the extension lines denote the extremes not tagged as outliers and the single standing plus marks as the outliers. The variance graph is plotted using the variance function which computes the square of the standard deviation. The correlation coefficients are calculated by the 2-d correlation coefficient function.

**Figure 7:**
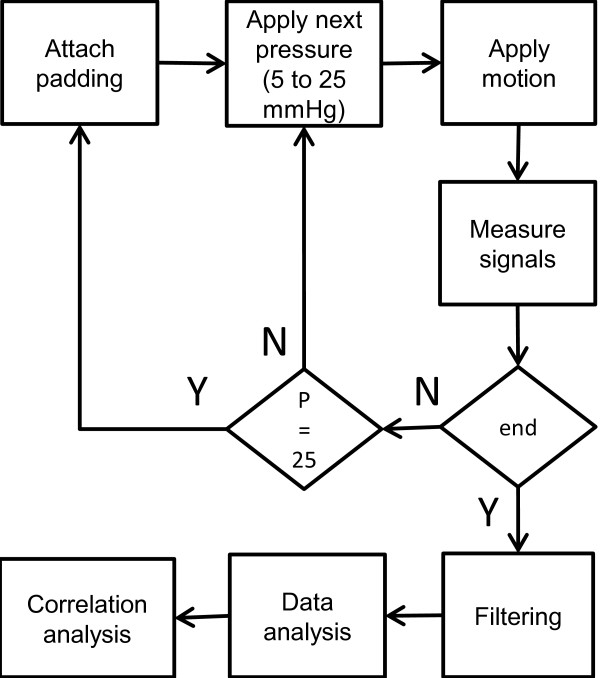
**Flowchart summarizing the methods.** The “end” decision box checks if measurement round 6 is finished. “P” is pressure and is incremented at each step by 5. After Padding 6 is measured, a new round starts with Padding 1. This loop is omitted for the sake of simplicity.

Figure 7 depicts a simple summary of the workflow.

## Results

As expected from the skin model presented in Figure 1 and the accompanying theory, electrode-skin interface impedance changes with motion. This specific motion causes an increase in the upper arm circumference, which simultaneously causes the skin to stretch and the pressure exerted by the elastic ribbon to increase. The motion artifact, measured as a surface potential, and shows a clear relation to motion.

**Figure 8:**
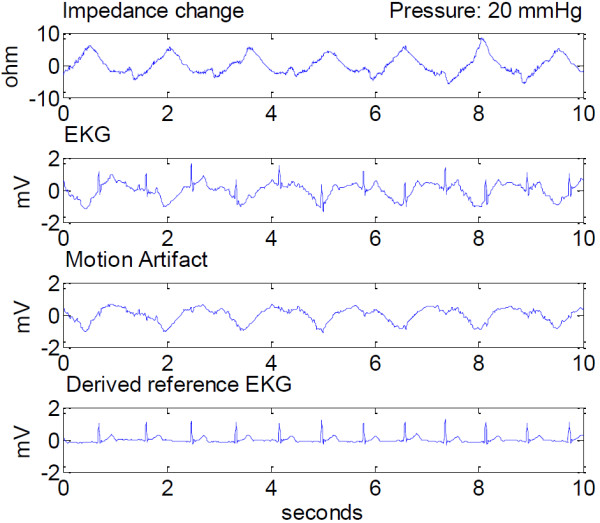
**Measured impedance change, ECG and motion artifact, and the derived chest ECG at 20 mmHg applied pressure.** Signals are presented after 0.3Hz–30 Hz band-pass filtering, and shown in a 10 second window.

An example of the observed impedance change, motion artifact as measured as a surface potential, and ECG with motion artifact, for a selected padding, for 20 mmHg pressure is shown in Figure 8.

**Figure 9:**
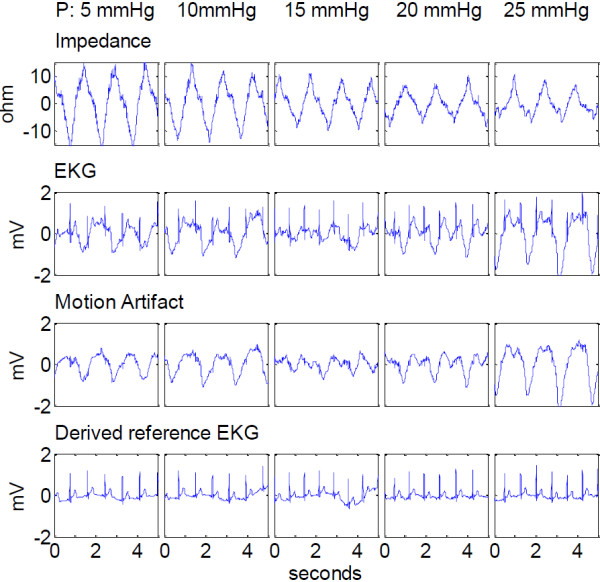
**Effect of increasing electrode pressure on the motion artifact related parameters.** From top to bottom: the real time impedance signal, the ECG, the motion induced surface potential (motion artifact) and the reference ECG derived from the electrodes on the chest. The corresponding pressure for each graph box increases from left to right, from 5 mmHg to 25 mmHg in 5 mmHg increments.

The effect of increased pressure on these signals is shown in Figure 9.

Correlations of the peak-to-peak values, the base impedance, the energy values between 1 Hz and 7 Hz as scores, the score from the R-peak detection, and applied pressure show that the electrode-skin interface impedance reduces with increasing pressure, and so does the peak-to-peak of the motion induced impedance change. The peak-to-peak of the motion artifact and the energy of the motion artifact between 1 Hz and 7 Hz seem not to be affected by the increasing pressure. As expected, the energies of the impedance and the surface potential closely follow the peak-to-peak values as the smaller, higher frequency components do not contribute much to the overall energies of the signals. These behaviours are shown in the following Figure 5, which is a compilation of the data from all the paddings. The data obtained from the case without using padding differs from the data obtained with the usage of paddings. As the latter data forms a comparison group, the case without padding is excluded. In this figure it is seen that the electrode scoring method from the modified R-peak detection algorithm shows that increasing the pressure increases the signal reliability up to around 20 mmHg exerted pressure. After this point the motion artifact increases and the signal to noise ratio decreases.

Looking closer at the R-peak detection algorithm results, in Figure 6, it is clear that various paddings have differing behaviour, yet for all cases but one, the optimal range for applied pressure is 15–20 mmHg pressure. Between these pressure levels, all the paddings provide higher signal reliability than using only a ribbon and a textile electrode, which would be the case in integrating the textile electrode directly into the ribbon or the garment. The paddings’ behaviours differ slightly, but the effect of increasing the pressure beyond an optimum level is seen to increase the motion artifact.

**Figure 10:**
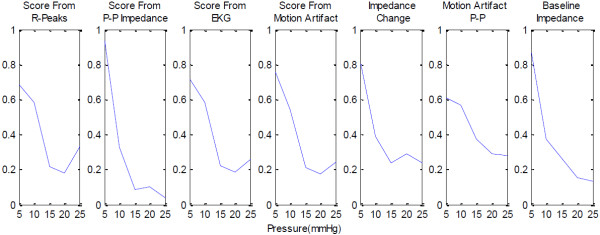
**Normalized variance graphs of various parameters, excluding the case of no padding.** The data is normalized using the Matlab function “norm”, with the formula: normalized data = data/norm(data).

The variance of the parameters decreases with increasing pressure. This effect is demonstrated in Figure 10, and shows clearly that all the values measured have less variance when pressure is increased.

Looking at the other correlations, a moderate correlation exists between the R-peak detection algorithm score and the energy of the motion artifact between 1 Hz and 7 Hz. This could be due to the used energy band including the higher frequency motion artifact components which are more detrimental on the ECG signal quality than the slower baseline shift (f < 1 Hz) which is not picked up by this R-peak detection algorithm.

Other noteworthy findings are that the base impedance and impedance change show a low correlation, the same relation exists between the peak-to-peak of the motion artifact and the base impedance and also between the energy of the motion artifact and base impedance. These correlations are listed in Table 1. Unlisted correlations between the parameters are in the low or non-existent levels.

**Table 1:** Correlation coefficients of selected parameter

**Parameters**	**Score from imp. change**	**Score from ECG**	**Score from motion art.**	**Motion art. peak-to-peak**	**Imp. change peak-to-peak**	**Baseline impedance**
Score from R-Peak detection		0.63	0.61	0.59		0.36
Score from imp. change						0.53
Score from ECG			0.99			0.41
Score from motion art.		0.99				0.44
Motion art. peak-to-peak						0.45
Imp. change peak-to-peak						0.50

## Discussion

In this study we investigated the behaviour of textile electrodes under various pressures exerted by placeholder ribbons and the effect of using paddings between the electrode and the ribbon. The real time electrode-skin interface impedance, ECG and the motion artifact, and pressure under the ribbon were measured. The electrode-skin interface impedance was measured from a two electrode setup. The motion artifact and ECG were measured by a novel method using two measurement channels which have the common reference electrode at the lateral upper arm location which was exposed to motion. One channel measured the motion artifact. The other channel measured the same motion artifact superimposed on an ECG signal. The motion artifact was created at the upper arm by flexing and extending elbow joint, synchronized to a metronome. The correlations between measured and calculated parameters were sought in order to provide guidelines on how to improve signal quality under motion and on to examine the possibility of estimating the electrode signal quality by measuring the real time impedance under motion. The variances in the data were investigated in order to understand the convergence of noise to a manageable level. In this paper an electrode scoring method concerning the motion artifact was devised by modifying the Pan-Tompkins QRS detection algorithm by removing error checks and refractory period considerations, and comparing the detected peaks of a noisy channel to a channel without motion artifact.

Six different measurements were done for each padding and pressure combination, totalling 180 measurements, adding up to 150 minutes of data. That these measurements were from one test subject may seem like a disadvantage, but we considered that increasing the repetitions of one subject would provide more applicable results to an initial study due to the large variability between subjects.

Our findings show that increasing the pressure on the electrode reduces the motion artifact, the variance of the measured signals, the electrode-skin interface impedance in relaxed state, and the changes in the real time impedance (100 kHz) due to motion, when using padding and when not using padding. The overall best effect is observed between 15 mmHg and 20 mmHg, after which, increasing the pressure proves to be detrimental to signal quality.

In the context of this study, previous studies have shown that increasing the pressure on the electrode lowers the electrode-skin interface impedance [32], causes a surface potential change [32,33] and this lowered impedance increases the signal quality at rest [34]. These studies were in line with the generally accepted skin model presented by Neuman and Webster [29], who states that the main component in the motion artifact is the potential across the epidermis, not the electrode-skin interface impedance [27]. Newer studies, though, have shown a direct correlation between the motion and the electrode-skin interface impedance. The effect of pressure and stretch applied on the skin for commercial electrodes [16,25] dry electrodes [9,18] and textile electrodes [19,35] have been studied, showing a direct relation. Contradictory results have also been published, showing no relation between the electrode-skin interface impedance and motion artifact [20]. These motion artifact studies were based on inducing motion by externally applied stretch or pressure to the skin.

Our measurements show that the electrode-skin interface impedance at rest, as well as the motion induced impedance change decreases with applied pressure. The amplitude of the motion artifact’s basic frequency component also decreases with increasing pressure. The same signal’s higher frequency components (1 Hz < f < 30 Hz) show a larger dependency to the applied pressure than these slower frequency components that are present as a baseline wander. These higher frequency components of the motion artifact decrease up to 20 mmHg applied pressure and then increase with increasing pressure. With the higher frequency components of the motion artifact having a larger distortive effect on the ECG, this points to a sweet spot where the motion artifact is minimized with the given setup. In this range of 15 to 20 mmHg, the median false R-peak detection ratio is reduced by 35% and 65% compared to 10 and 25 mmHg pressures.

The variability of the measured parameters decreases with increased pressure. This decrease is between 70% and 90% for different parameters. While not directly minimizing, or eliminating the motion artifact, this reduction in the variability of the measurements renders the motion artifact to become more predictable, which aids in minimizing its effects on the ECG. The decrease in the variability shows the same characteristics as the above mentioned decrease in the motion artifact and in the changes of real time impedance. A sweet zone between 15 mmHg and 20 mmHg applied pressure is observed also for variability.

Regardless of applied pressure, using paddings considerably improves signal quality and lowers motion artifact. In most cases, the motion artifact shows correlation to the changes observed in the electrode-skin interface impedance (100 kHz) during motion. In a minority of the trials, the motion artifact shows little or no relation to the motion induced changes in the electrode-skin interface impedance. This is especially true when the impedance change contains only low frequency components (f < 1 Hz), while the motion artifact contains higher frequency components, without the baseline wander. For this issue, the padding material choice seems to be of importance; a subject for further studies investigating the padding size, shape, dynamic material properties and also location dependence in relation to the motion artifact.

Our results show that the skin and the electrode-skin interface impedance at 100 kHz changes with motion, but this change is not enough to explain the motion artifact as a whole. This can be explained by the epidermis potential being the dominant factor in the motion artifact for some cases, instead of the electrode-skin interface. One drawback of the study is that the skin and the electrode-skin interface impedance at 1 Hz–100 Hz might have different reactions to motion than impedance at the measured 100 kHz. Nevertheless, factors that lower the electrode impedances are still advisable for the reduction of the motion artifact: In the case where there is a relation between the electrode-skin interface impedance and the motion artifact, lowering the impedance will decrease the motion artifact; in the case where the motion artifact is not related to the electrode-skin interface impedance, the reduction of the impedance will not decrease the motion artifact but then again it will have no negative effect either. This difference in behaviour can be seen even at the same electrode-padding combination, which may behave inconsistently, concerning the relation of motion artifact with impedance.

## Conclusion

We have devised a novel two lead method using three electrodes for the estimation of the motion artifact on ECG and a method to generate repeatable motion artifact. This method utilizes an R-peak detection algorithm modified from the Pan-Tompkins QRS detection algorithm to quantify the artifact. The method quantifies effects of used padding and applied pressure on the motion artifact and has given the possibility for providing the following design guidelines for electrode assembly: A padding, to be used between the attachment medium and the conductive textile electrode material, increases the skin contact quality and stabilizes the electrode against motion. The attachment method should exert a pressure between 15 to 20 mmHg to provide the best signal quality and best stability against motion artifact, due to the stabilization of the electrode on the skin and due to the electrical properties of the skin under that level of pressure, which coincidentally falls into the tight but still comfortable range.

## Abbreviations

ECG: Electrocardiogram; EMG: Electromyogram.

## Competing interests

The authors declare that they have no competing interests.

## Authors’ contributions

AC: designed the experiment, conducted the experiment, wrote the manuscript. MH: material acquisition and advice related to materials and electrode structures, reviewed the manuscript for scientific content. JH: helped with study design and experiment set up design, guidance in data interpretation, reviewed the manuscript for scientific content. All authors read and approved the final manuscript.
